# Expression of Endogenous Retroviral RNA in Prostate Tumors has Prognostic Value and Shows Differences among Americans of African Versus European/Middle Eastern Ancestry

**DOI:** 10.3390/cancers13246347

**Published:** 2021-12-17

**Authors:** Vinay Kumar, Michael McClelland, James Nguyen, Gabriela De Robles, Michael Ittmann, Patricia Castro, Dan Mercola, Zhenyu Jia, Farah Rahmatpanah

**Affiliations:** 1 Department of Pathology and Laboratory Medicine, University of California, Irvine, CA 92697, USA; vkumar4@uci.edu (V.K.); mmcclell@uci.edu (M.M.); jamesan3@hs.uci.edu (J.N.); gderoble@uci.edu (G.D.R.); dmercola@uci.edu (D.M.); 2 Department of Microbiology and Molecular Genetics, University of California, Irvine, CA 92697, USA; 3 Department of Pathology, Baylor College of Medicine, Houston, TX 77030, USA; mittmann@bcm.edu (M.I.); pcastro@bcm.edu (P.C.); 4 Department of Botany and Plant Sciences, University of California, Riverside, CA 92521, USA; zhenyuj@ucr.edu

**Keywords:** human endogenous retrovirus, prostate cancer, cancer prognostics, genetic variation, ancestral differences

## Abstract

**Simple Summary:**

Endogenous retroviruses (ERVs) are viral sequences that have been incorporated into the human genome over millions of years via integrations in germ-line cells. In this study, we investigated whether the expression of ERVs was associated with two different aspects of prostate cancer (PCa). First, Black American men have a higher incidence and poorer outcome of PCa compared to White men. We identified differences in ERV expression among prostate tumors between men of primarily African versus primarily European or Middle Eastern ancestry, which may be associated with differences in the mechanism of cancer progression in patients of these distinct ancestries. Second, we determined whether ERV expression might be correlated with the progression of disease, regardless of ancestry. We identified the ERV expression signatures that correlated with biochemical relapse among PCa patients of all ancestries, indicating that ERVs may be useful for identifying cancer patients at greatest risk of progression. The utility of ERV expression for studying cancer progression may extend to other cancers.

**Abstract:**

Endogenous retroviruses (ERVs) are abundant, repetitive elements dispersed across the human genome and are implicated in various diseases. We investigated two potential roles for ERVs in prostate cancer (PCa). First, the PCa of Black Americans (BA) is diagnosed at an earlier median age and at a more advanced stage than the PCa of White Americans (WA). We used publicly available RNA-seq data from tumor-enriched samples of 27 BA and 65 WA PCa patients in order to identify 12 differentially expressed ERVs (*p*_adj_ < 0.1) and used a tissue microarray of the PCa cores from an independent set of BA and WA patients to validate the differential protein expression of one of these ERVs, ERV3-1 (*p* = 2.829 × 10^−7^). Second, we used 57 PCa tumors from patients of all ancestries from one hospital as a training set to identify the ERVs associated with time to biochemical relapse. A 29-ERV prognostic panel was then tested and validated on 35 separate PCa tumors from patients obtained in two different hospitals with a dramatic increase in prognostic power relative to clinical parameters alone (*p* = 7.4 × 10^−11^). In summary, ERV RNA expression differences in the prostate tumors of patients of different ancestries may be associated with dissimilarities in the mechanism of cancer progression. In addition, the correlation of expression of certain ERVs in prostate tumors with the risk of biochemical relapse indicates a possible role for ERV expression in cancer progression.

## 1. Introduction

Prostate cancer (PCa) affects millions of men worldwide and remains the most common cancer diagnosed in men in the United States [1]. Many of these cancers are ultimately not the proximate cause of death [2]. However, the percentage of aggressive PCa is dissimilar between men of different ancestries. There is a higher prostate cancer burden among Black American (BA) as compared to White American (WA) patients [3,4]. Being Black American is an independent predictor of disease relapse in patients undergoing radical prostatectomy [5,6]. Recent studies indicate that larger numbers of PCa patients of African descent assigned to active surveillance undergo aggressive treatments within five years due to disease progression when compared to patients of European descent who are afflicted with the disease [7]. The correlations between the genetic factors and ethnic disparities in cancer, including PCa, have been uncovered and studied [4,8,9,10,11,12], occasionally focusing on gene expression differences in the PCa tumors of men of different ancestries [8,13,14,15].

Human endogenous retroviruses (ERVs) are viral sequences that have been continually incorporated into the mammalian genome over millions of years via new integrations in germ-line cells. ERVs constitute approximately 8% of the human genome, whereas protein-coding genes only represent 1–2% of human DNA [16,17]. However, few studies have focused on the impact of ERVs on human health and disease. Understanding how each ERV modulates both itself and other components, such as functional proteins, antigens, or regulatory elements, is necessary in order to characterize how the ERVs affect human disease [17,18].

Long terminal repeats (LTRs) of ERVs serve as genomic regulators and affect the transcription or splicing of nearby genes [17,19,20]. ERVs are silenced through DNA methylation and histone modifications, and while the majority of ERVs are functionally inactive, many ERVs can be activated after removing repressive epigenetic markers [18]. They have been implicated in autoimmune disease pathogenesis [21], but their immunogenic properties may play a beneficial role in cancer therapy by eliciting an immune response when activated in cancers [22]. The analysis of high-throughput sequencing data in The Cancer Genome Atlas suggests that patients with high expression levels of ERV transcripts and antiviral immune response genes have a more favorable prognosis and benefit more from immune therapy than those lacking the expression of these genes in their tumor cells [22,23,24].

Here, we investigated expression among intact and autonomous ERVs in a curated collection of 3220 ERVs from the ERVmap database [17], which were applied to published RNA-seq data from 92 tumor-enriched PCa samples (GSE54460 [1]) (27 BA and 65 WA). We also separately analyzed the expression of all of the other host genes and identified those that were adjacent to the ERVs. Of the ERV-adjacent genes, we identified those that had concordant regulation to the ERVs in both the BA and the WA patients separately.

Most of the ERV sequences have acquired numerous mutations over time and, therefore, do not have protein-coding potential nor the capability to generate infectious viral particles [17]. However, a few of the ERVs retain a complete open reading frame, allowing the quantification of their protein expression in tissue samples. We used PCa tissue microarrays (TMAs) from 142 independent PCa patients of two different ancestral groups (i.e., 52 BA and 90 WA) in order to characterize the protein expression of ERV3-1. This ERV was chosen because it appears as one of the twelve most significantly differentially expressed ERVs and is one of the small minorities that retains its protein-coding capability.

ERVs can be useful as prognostic biomarkers [25,26,27,28]. Therefore, we looked at the association between ERV expression and the risk of biochemical relapse (BCR). By using the same RNA-seq data as above, we employed a subset of samples from one hospital (regardless of ancestry) as a training set to identify a panel of predictive ERVs using a Lasso-Cox proportional hazard model [29]. We show that when the resulting 29-gene ERV expression prognosticator is applied to a separate test set of patients from two other hospitals, the risk assessment for relapse greatly improves compared to the clinical parameters alone.

## 2. Materials and Methods

### 2.1. Sample Selection

Raw RNA-seq data of tumor epithelium from 106 PCa samples were obtained from the gene expression omnibus (GEO) database (GSE54460), accompanied with complete clinical data [1]. A total of 6 duplicates were identified and subsequently removed from further analysis. The specimens were collected from three different hospitals (Atlanta Veterans Affairs Medical Center at the Emory School of Medicine, Decatur, VA, USA, Sunnybrook Health Sciences Center at the University of Toronto, and Moffitt Cancer Center in Tampa, Tampa, FL, USA).

### 2.2. Locating Ancestry from Sequence Reads (LASER)

In order to identify the preponderant geographical ancestry of each patient, we used LASER, which estimates individual ancestry by directly analyzing sequence reads without calling genotypes [30]. LASER places each sample into a reference principal component analysis space constructed with 632,958 SNPs of reference individuals, and the estimated coordinates of the sequence samples reflect their ancestral background. The reads were first aligned to the NCBI hg19 build (hs37d5.fa) using STAR [31]. Using the SAMtools [32] *pileup* command and the genomic regions BED file provided alongside the LASER executable, we generated pileup files containing annotated regions from the alignments [30,33]. Using the LASER pipeline, we then predicted the ancestry of each patient by allowing the program to compute 3 principal components for the reference panel and 20 principal components for the projection of the study samples. LASER verified the primary geographical ancestry of the patients and imputed the primary ancestry of patients lacking ancestry information in the dataset (GSE54460), as previously described [34].

### 2.3. RNA-Seq Analysis of ERVmap

Following the LASER analysis, patients of primarily Asian ancestry (*n* = 4) and samples of low read quality (*n* = 4) were removed prior to the alignment, resulting in 92 overall patient samples to be analyzed. We developed a pipeline to analyze and count reads for ERVs found in the ERVmap [17] database, which contains 3220 autonomous and recently incorporated ERVs, identified in the context of different diseases [17]. Briefly, raw reads were first trimmed and checked by Trimmomatic [35] to remove the Illumina adapters. The trimmed reads were then aligned to the human genome reference sequence (hg38) with STAR [31] using a strict mapping criterion to ensure that reads were counted only when they mapped uniquely to one location. The ERVmap database contains only genomic region annotations for ERVs with start and end loci. The BAM file containing the alignments, together with a BED file containing the ERVmap were used as inputs for the BEDTools [36] *coverage* function to generate the number of reads mapping at each ERV locus, thus producing the raw expression data for the ERVs.

### 2.4. Transcriptome Analysis of ERVs in Black American and White American PCa Patients

In order to determine ERV expression differences between BA and WA PCa patients in the tumor samples [1], raw read counts for the regions provided by ERVmap were imported for use by *DeSeq2* [37]. Normalization of the ERV read counts was done by applying the size factors from the cellular gene expression of the same samples. Briefly, a GTF file containing the gene annotations from Ensembl was provided as an additional parameter to the previously mentioned STAR pipeline to produce the raw counts for the cellular genes. *DeSeq2* was used to calculate the size factors after collecting the read counts. The size factors were then applied to the raw ERV counts to generate the normalized expression counts, which were then provided as an input for the differential expression pipeline.

### 2.5. ERV-Targeted Genes

ERVs may modulate the activity of nearby genes [38,39,40]. Adjacent genes located within 5000 bp of each significantly differentially expressed ERV (*p*_adj_ < 0.1) were identified using the BEDtools [36] *closest* function. Two inputs were provided, as follows: a filtered ERVmap BED file containing ERVs with significant differential expression between BA and WA patients and a BED file containing the gene annotations from Ensembl.

### 2.6. PCa Tissue Microarrays and Immunohistochemistry

Prostate cancer tissue microarrays (TMAs) of 54 self-reported BA patients and 106 self-reported WA patients were constructed at the Baylor Department of Pathology and Immunology using surgical radical prostatectomy resected specimens (https://www.bcm.edu/research/atc-core-labs/human-tissue-acquisition-and-pathology (accessed on 14 December 2021).

Blocks from each TMA were stained with primary antibody for ERV3-1 (Thermo Fisher CAT # PA5-48577, 1:200) by Crown Bioscience, Inc. Immunohistochemistry was performed on the Bond RX autostainer (Leica Biosystems) by a heat-induced epitope retrieval treatment in citrate buffer at pH 6.0. Bond Polymer Refine Detection (Leica Biosystems, DS9800) uses a secondary antibody according to the manufacturer’s standard protocol. After staining, sections were dehydrated, and films were covered using a Tissue-Tek Prisma and Coverslipper (Sakura, Japan). Slide scanning was performed on a NanoZoomer Digital Slide System NDP2.0-HT (Hamamatsu, Japan).

### 2.7. Statistical Analysis of ERV Protein Expression in TMAs from BA and WA PCa Patients

From the original set of 160 TMA samples, 18 cores that lacked an appropriate amount of tissue were excluded for ERV3-1. As a result, 52 tumor tissue cores from BA and 90 from WA patients were quantified using QuPath [41]. Positive pixel counts were computed for each individual core to quantify the amount of stained tissue on the slides. Percent positivity was calculated by dividing the number of positive pixels by the total number of pixels identified for the stain, excluding non-stained background regions. A student’s t test was used for comparison between the BA and WA tissue microarray data. A *p*-value < 0.05 was considered significant.

### 2.8. Selection of ERV Biomarkers for Prognostication of Biochemical Relapse

Normalization of the raw ERV expression counts produced from GSE54460 [1] was performed by using the size factors obtained from standard cellular gene expression analysis and processing in the *DeSeq2* [37] package as previously described. A variance-stabilizing transformation (z-score) was then applied to the normalized expression data for downstream analysis. Samples from the Atlanta VA Medical Center (*n* = 57) were selected as the training set to generate the initial ERV panel. A preselection step was completed by providing individual ERV expression and survival data as the parameters for a univariate Cox model. ERVs that passed a FDR threshold of 0.10 were selected as inputs to a Lasso-Cox proportional hazards model, along with time to relapse and event status using the *glmnet* [42] package. This model was used to identify ERV markers that are significantly associated with biochemical relapse time, where the tuning parameter for the lasso penalization was selected using a leave-one-out cross-validation technique. An internal 10-fold cross-validation confirmed the selected ERV markers.

### 2.9. Risk Evaluation

A risk-prediction model was built using ERV expression data from the panel of selected ERVs + clinical data, including Gleason score, tumor stage, age, and prostate-specific antigen (PSA), by fitting a Cox proportional hazards model. Gleason scores were divided into four categories based on the Epstein Grade group [43] prior to model creation. The PSA data was log-transformed by log_(PSA+1)_ to avoid skewing by extreme outliers. To generate the initial risk assessment model, samples from the Atlanta VA Medical Center (*n* = 57) were once again selected as the training set. Samples were then assigned to high- and low-risk score groups based on whether the linear predictors from the Cox proportional hazards model were greater (higher risk) or less (lower risk) than 0. Another risk model, consisting of only the clinical data (Gleason score, tumor stage, age, and PSA), was produced using the same procedure mentioned above.

The test set for this model consisted of a combined set of patients (*n* = 35) from two different hospitals, the Moffitt Cancer Center and the University of Toronto Sunnybrook Medical Centre. The trained model was applied to the data from the test set, using both the predictors from the model made with the ERV panel + clinical data and the model made using only the clinical data.

To compare the performance of the model created with the ERV panel and clinical data with that of the clinical data alone, the following three measures were used: median improvement in risk score, integrated discrimination improvement, and net classification improvement [44].

### 2.10. Survival Model

As previously mentioned in Section 2.8, the ERVs first underwent a preselection step through a univariate Cox proportional hazards model. The list of ERVs that survived were used as inputs for a penalized Lasso-Cox proportional hazards model, which produced a group of ERVs that, together, had significant prognostic value in determining recurrence.

The group of ERVs selected from the lasso model was provided as an input for a multivariate Cox proportional hazards model alongside the aforementioned clinical data. This model produced linear predictors that separated samples into a binary classification of either high or low risk.

This risk evaluation and the time to recurrence were provided as inputs to the *survfit* function in the *survival* R package to generate the Kaplan–Meier model. Plotting of the survival curve was done by using the *survfit* function and the *plot* function present in the R standard base library.

## 3. Results

### 3.1. ERV Expression Profiles of Tumor-Enriched Samples from Black and White American PCa Patients

**Figure 1:**
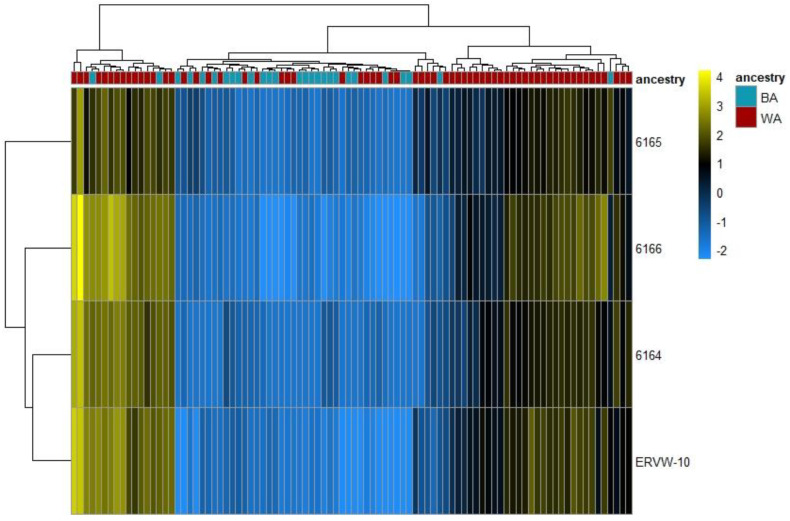
Four most differentially expressed ERVs among Black and White Americans. ERVs from 92 tumor-enriched PCa samples were mapped to the ERVmap database. Differential expression analysis and hierarchical clustering analysis (Euclidean) was performed in *DeSeq2* to organize tumor samples based on their ERV transcription profiles and the ancestry of the PCa patients. The heat map represents the hierarchical clustering of the four significantly differentially expressed ERVs (*p*_adj_ < 0.1) and had a |log_2_(FC)| > 2 in BA (*n* = 27) vs. WA (*n* = 65). Each column represents one sample and each row represents a single ERV. The ERV expression is depicted as a color intensity (−2.0 to 4.0) on a log scale; yellow indicates ERVs with higher expression and blue indicates lower expression. Expression was normalized to the mean of all samples. In the dendrogram on top, the ancestral origin of each patient is indicated by color. Red indicates patients of predominantly European (*n* = 54) or Middle Eastern (*n* = 11) geographical ancestries while blue represents patients of African ancestry.

**Figure 2:**
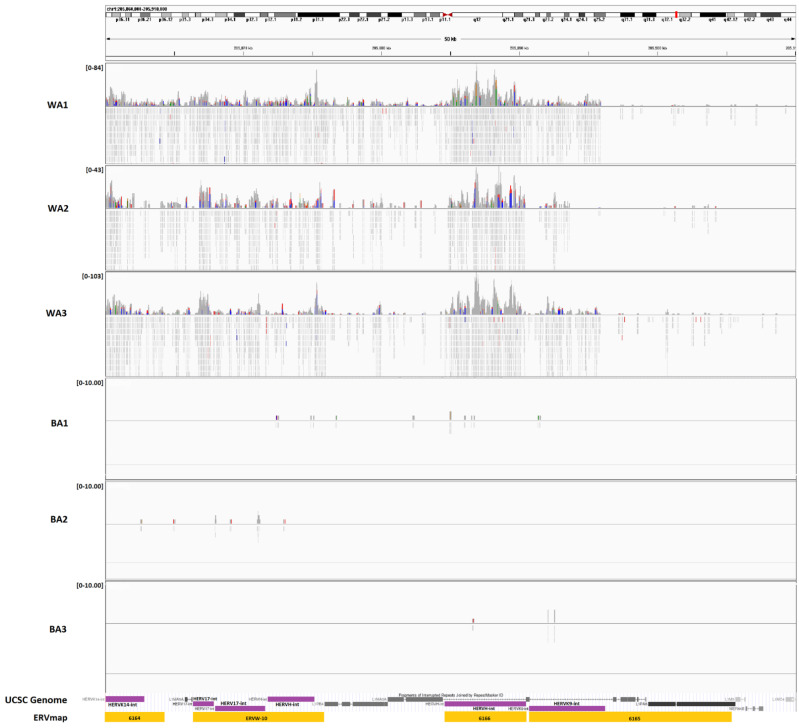
Representative tracks of RNA-seq expression data for chr1:205,860,000-205,910,000 containing ERVs 6164, 6165, 6166, and ERVW-10, which are members of the ERVK and ERVW families, visualized using the integrated genomics viewer (IGV). Read coverage for WA depicts a high level of dissimilarity from BA patients in the same region. The purple annotations represent canonical ERV names according to the RepeatMasker UCSC genome browser track while the yellow represents their corresponding annotations in the ERVmap database.

In order to explore whether the ERV expression varies among PCa patients with different geographical ancestries, we analyzed the tumor-enriched RNA-seq data from GEO GSE54460 [1] (Tables S1 and S2). Of the 92 samples used, ancestral breakdowns, confirmed by LASER analysis, were as follows: 27 of primarily African ancestry, 54 of primarily European ancestry, and 11 of primarily Middle Eastern ancestry. We classified those of Middle Eastern ancestry (*n* = 11) and European ancestry (*n* = 54) under an aggregate classification of the White cohort (*n* = 65) because population studies using 1000 genomes revealed a smaller genetic distance between Middle Eastern and European ancestries when compared to those of African descent [45,46]. After the alignment, differential expression analysis was performed on the count data for the ERVs. Of the 3220 autonomous, full length ERVs from the ERVmap, we identified 12 with a significant differential expression (*p*_adj_ < 0.1) (Table S3), four of which had a |log_2_(FC)| > 2 and were upregulated in WA versus BA patients (Figure 1). Among the four with a high fold-change difference were members of the ERVK (ERVK9 and ERVK14) and ERVW (W-10) families, and all were clustered within 50kbp of each other on chromosome 1 (chr1: 205,860,000-295,910,000) (Figure 2). ERV3-1, which contains an open reading frame and can be studied through protein expression analysis, was also among the top 12 most significant ERVs identified.

### 3.2. Protein Expression Analysis of ERV3-1 in PCa of BA and WA Patients Using TMAs

**Figure 3:**
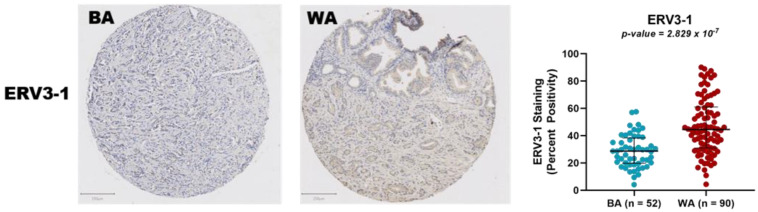
Tissue microarray slides stained with the ERV3 antibody. A tumor tissue sample from a patient of BA ancestry is shown on the left while a sample from a patient of WA ancestry is shown on the right. A scatter plot of ERV3-1 percent positivity in tissue microarrays (TMA) from 52 BA and 90 WA patient samples is also shown on the right. Blue represents BA while red represents WA.

Twelve ERVs from the ERVmap were differentially expressed at the RNA level between the BA and WA patients, including ERV3-1, which was expressed at a significantly lower level (*p*_adj_ < 0.1) in the BA patients than in WA patients (Figure 3, Table S3). In order to determine if differential expression of ERVs between people of different ancestry was also manifested at the protein level, we further investigated ERV3-1. This ERV was selected because it is not only one of the twelve most significant (*p*_adj_ < 0.1) in the RNA-seq analysis, but also one of few ERVs that retains an open reading frame, allowing for the investigation of its protein expression. An ERV3 antibody was applied to a large tissue microarray of tumor tissue from an independent set of BA (*n* = 52) (Table S4) and WA (*n* = 90) (Table S5) patients, and protein production was scored as percent positive pixels using QuPath, as described. Representative stains of both tissue microarrays can be found in Figure 3. As was consistent with the RNA expression data, we found significant differences in the protein expression for ERV3-1 (*p* = 2.829 × 10^−7^, Figure 3).

### 3.3. Expression of Genes Adjacent to Differentially Regulated ERVs

Numerous studies suggest that LTR elements of ERVs may exert regulatory functions on nearby genes [47,48]. ERVs serve as cis and trans transcriptional regulators, alternative promoters, polyadenylation signals for several cellular genes, and splice donors or receivers [17]. ERVs also contain binding sites for transcriptional factors, such as STAT1 and p53, which may regulate the expression of their nearby genes [17,39,48,49]. As an example, in the CRISPR-Cas9 deletion of four different individual LTR elements in the MER41 family of ERVs in the human genome, each impaired expression of an adjacent interferon-stimulated gene [48]. We therefore looked for coordinated expression of adjacent genes among ERVs that were differentially expressed between the BA and the WA PCa patients.

Among the nearby upregulated genes, in concordance with ERVs (i.e., MER61) in WA patients, was TP53TG1. Studies have shown that this gene plays an important role in the regulation of various cancers, inducing cell proliferation and inhibiting apoptosis in pancreatic ductal adenocarcinomas, while inducing apoptosis and increasing sensitivity to cisplatin in non-small cell lung cancer [50,51]. One of the other nearby genes found to be upregulated in the WA patients and the nearby significantly different ERVs (ERV #6164, ERV #6165, ERV #6166, and ERVW-10) was lncRNA ribosomal protein 4 (RP4). This gene has been identified to have a role in cell proliferation, tumor growth, and early apoptosis in colorectal cancer cells [52]. The combination of the regulatory effects and cancerous associations suggests a link between the expression of these elements among patients of different ancestries.

Of the nearby genes with differential expression downregulated in the BA patients, in the same direction as the ERVs, was haptoglobin-related protein (HPR). HPR is a high-affinity hemoglobin-binding plasma protein that has been shown to have an association between its expression, breast cancer malignancy, and recurrence [53]. Furthermore, it has been identified as a possible serum marker of lymphomas [54,55].

### 3.4. Biochemical Relapse Risk Assessment of Selected ERVs in PCa Patients

Classifying patients into those that are at low risk and those that are at higher probability of disease progression is of importance for patient management. Although Gleason score, a measure of cellular abnormality, remains an important indicator of disease progression, clinical parameters alone have shown limited utility in predicting the risk of biochemical relapse (BCR) after prostatectomy [56]. Fortunately, supplementation of clinical parameters with gene expression data can strengthen the accuracy of a risk assessment. In a 2014 study, Long et al. identified 24 genes that are differentially expressed in PCas of dissimilar BCR risk, which can be utilized to enhance the prediction precision and thereby improve the clinical management of prostate cancer [1]. Similar analyses focusing on ERV expression within PCa samples, and its possible association with BCR, have not been performed to date.

We built a training model using only samples from the Atlanta VA Medical Center, regardless of ancestry (*n* = 57), and a testing model using patients (*n* = 35) from the two other hospitals, the Moffitt Cancer Center and the University of Toronto Sunnybrook Research Centre (Table 1).

**Figure A1:**
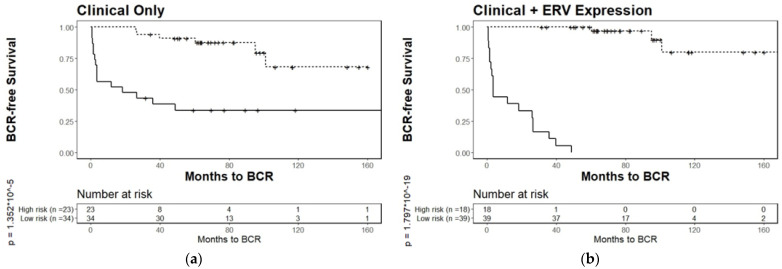
Kaplan–Meier plots for biochemical relapse of a training set of PCa patients stratified by prognostic models based on (**a**), clinical data alone, or (**b**), clinical data + 29-ERV panel. Tumor-enriched samples from PCa patients from the Atlanta VA Medical Center (*n* = 57) were separated into two risk groups based on biochemical relapse. The prognostic model applied in (**b**) (*p* = 1.797 × 10^−19^) outperforms clinical parameters alone, in (**a**) (*p* = 1.352 × 10^−5^), and consists of 29 ERVs, identified using survival analysis based on a Lasso-Cox proportional hazard model, combined with the same clinical variables used in model (**a**).

After a preselection step using univariate Cox analysis, the expression levels of 3220 ERVs from samples in the training set were used to build a 29-ERV prognosticator of BCR using a Lasso-Cox proportional hazards model, and the final risk-prediction model was built by combining this panel of 29 markers with the available clinical data, including Gleason value, PSA, age, and tumor stage (Table S6). For comparison, we also built a prediction model using only the previously listed clinical variables by fitting a Cox proportional hazards model. The linear predictors from both models separated patients into high- and low-risk groups (Appendix A, Figure A1).

Kaplan–Meier survival analysis, using only clinical parameters, reached a significance level of *p* = 1.3252 × 10^−5^ (Figure A1a). The full model, which incorporated the 29-ERV panel and clinical data, discriminated the two risk groups of patients for time to BCR at a much higher significance level of *p* = 1.797 × 10^−19^ (Figure A1b). The 29-ERV panel was internally validated by a 10-fold cross-validation performed by the *glmnet* [42] package when creating the Lasso-Cox proportional hazards model [29].

**Figure 4:**
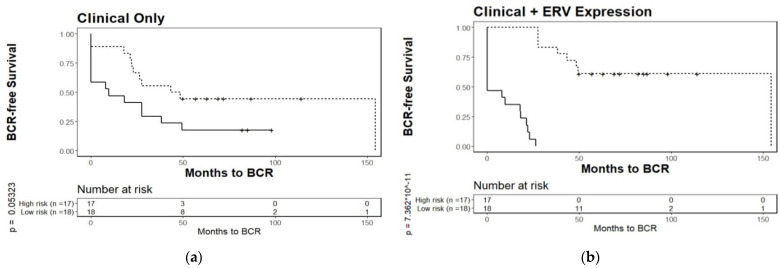
Kaplan–Meier plots for biochemical relapse of a test set of patients stratified by prognostic models based on (**a**), clinical data alone, or (**b**), clinical data + 29-ERV panel obtained from a training set. Tumor-enriched samples from PCa patients from the Moffitt Cancer Center and the University of Toronto Sunnybrook Medical Centre (test set of *n* = 35, originating from different hospitals than those used for the training set) were separated into two risk groups for biochemical relapse. The prognostic model applied to the test set in (**b**) (*p* = 7.362 × 10^−11^) significantly outperforms clinical parameters alone, in (**a**) (*p* = 0.05323).

External validation with a separate test set of patients was done by using a combined set (*n* = 35) of patients from the two other hospitals, the Moffitt Cancer Center and the University of Toronto Sunnybrook Research Centre. Briefly, the linear predictors created from the training set were applied to each patient from the test set. Subsequently, subjects were divided into BCR risk groups. The clinical model alone had a marginal prognostic value in the test set (*p* = 0.05323) (Figure 4a), while a significant prognostic value was achieved in the combined panel of ERV biomarkers and clinical variables (*p* = 7.362 × 10^−11^) (Figure 4b). The improvement of the full model over the clinical model was further assessed by using the median improvement in the risk score (score: 0.591, *p* < 0.001), integrated discrimination improvement (score: 0.859, *p* < 0.001), and net classification improvement (score: 0.472, *p* < 0.001) [44]. Based on the ROC, the AUC value for the ERVmap model was 0.878.

We also performed a 10-fold cross-validation, in which all 92 samples, from both hospitals, participated in one of ten non-overlapping test sets. In each of the ten validations, 10% of the samples were chosen for the test set and the remaining 90% were selected as the training sets. This analysis yielded a *p*-value of 3.077 × 10^−16^, indicating that the prognostic value of the biomarkers was widely present among the 92.

## 4. Discussion

Prostate cancer accounts for more than 34,000 deaths per year among males in the United States [1,57] with a higher incidence and greater mortality in Black Americans (BA) as compared to White Americans (WA) [3,58]. The differences in the rate and progression of diseases between people of different geographical ancestry have been associated with variations in gene expression [11,12,13]. However, the association between global ERV expression and geographical ancestry has, to date, not been investigated in PCa.

One barrier to investigating gene regulation by ERVs is the difficulty in the mapping of transcripts of the highly repetitive ERV families to unique loci when using the paired-end 100 bp and 150 bp reads typical of Illumina sequencing data. We developed a pipeline to work around these technological barriers, where instead of allowing the reads to map to all of the possible genome locations, we force them to map to a single best location. If no singular genome location can be identified as best match, the read remains unmapped and is not further analyzed. In the future, if the cost of generating longer reads falls, the yield of uniquely mapped reads will increase as more SNPs in each read allow for more frequent unique mapping.

To identify the expression levels of unique ERVs, we used the locations provided in the ERVmap database, which contains autonomous, more recent ERVs that are more likely to be transcribed [17]. When ERV expression in the PCa tumor samples of the 27 BA patients was compared to the 65 WA patients using the ERVmap database, some members of the ERVK, ERVW, and ERVH families were expressed at significantly different levels in the BA versus the WA patients (downregulated in BA). MER61, and its nearby gene TP53TG1, were also identified as downregulated. In a study by Wang et al., it was revealed that MER61 and LTR10 contain p53 binding sites, which impact the transcriptional network of human tumor suppressor protein p53 [59]. This suggests a critical role for ERV p53 sites in the direct regulation of p53 target genes. The HERVK-env (envelope protein) family of ERVs are among a small number of ERVs with protein-coding potential due to their open reading frame. Although controversial, studies suggest that human ERVK expression can stimulate the innate and adaptive immune response in breast cancer patients [28] where HERVK acts as a tumor-associated antigen, activating cytolytic T cells (CTLs), interferon production, and T helper 1 cytokine responses [28]. We identified multiple ERV loci with significantly different (*p*_adj_ < 0.1) expression rates at a high fold-change difference (|log_2_(FC)| > 2), containing multiple ERVK elements, that were upregulated in the WA patients over the BA patients (Figure 1).

In addition, ERV3-1 was found to be one of the twelve most significantly downregulated ERVs at the RNA level (*p*_adj_ < 0.1) in these 27 BA patients versus the 65 WA patients (Table S3). ERV3-1 is also one of the few ERVs with an open reading frame, which is why it was chosen for validation in the tissue microarray analysis. We probed PCa tissue microarrays from an additional 52 BA and 90 WA patients with an antibody directed to this protein, which revealed the same direction and magnitude of differential expression as seen with RNA (*p* = 2.829 × 10^−7^) (Figure 3). Furthermore, the biological value of ERV3-1 has also been seen in its possible tumor-suppressing capabilities in Hodgkin’s lymphoma patients and upregulation in prostate cancer patients [60]. This observation, in two independent datasets, reinforces the possibility that the expression differences between BA and WA ancestries may play a role in predisposition to disease and in the immune response to tumors [60,61]. Our data indicates that these ancestral predispositions may be linked to ERV differences.

Among genes and ERVs that were within 5000 bp distance from each other, and both significantly differentially expressed in the same direction (concordant), we identified two genes that played a role in various cancer types, TP53TG1 and RP4. The TP53TG1 gene has been proven to have both cancer inhibiting and cancer promoting effects in different cancer types [50,51]. The close proximity to the significantly differentially expressed ERVs lends credence to the idea that ERVs may have a regulatory effect on cancer related genes and possibly, between individuals of different ancestral backgrounds.

About 25–50% of men diagnosed with prostate cancer have low-grade disease, based on the PSA levels and clinical staging of prostate biopsies, and the majority of these patients are assigned to active surveillance without aggressive procedures, such as prostatectomy [6]. Gleason score and other clinical parameters, including age and tumor stage, are important indicators of disease progression and help to predict which patients can remain on active surveillance and which patients need further intervention, even after prostatectomy. However, prognostication using these markers remains imprecise, and the addition of other biomarkers, such as expression signatures to elevate prediction accuracy, is highly desirable [1].

As ERVs have been shown to have an underlying difference in expression levels in PCa patients of different ancestries, which are known to differ in the risk of cancer progression, we investigated whether ERVs might also predict BCR. Using the same publicly available RNA-seq data from tumor-enriched samples from PCa patients (GSE54460) [1] that was used here to identify expression differences in the ERVs between patients of varying ancestries, we selected a training set of samples (*n* = 57) from patients at the Atlanta VA Medical Center, while the other hospitals were assigned to the test set (*n* = 35). We built a panel of 29 ERVs potentially prognostic of BCR, using a Lasso-Cox proportional hazards model. A final risk-prediction model was built using the scores based on this panel combined with the clinical parameters. The 29-ERV signature in combination with the clinical data was a significantly better risk predictor than the clinical data alone, when validated on the separate test set (Figure 4). The ERV hazards ratio suggest that upregulation of specific ERVs results in increased risk for recurrence, while others may result in a lower overall risk (Table S6).

Of the 29 ERVs in the prognosticator, 25 contained elements (ERV1, HERVH-int, and ERVL–MaLR) that were previously shown to play a role in cancer [62,63] and four (ERV #1875, ERV #5137, ERV #5270, and ERVK-10) contained ERVK elements, which may play a role in prostate cancer patients [26,64,65]. Further investigation of other ERVs may reveal the effects of ERVs in various disease contexts and their prognostic effects. As RNA-seq data becomes more abundant in healthcare, so does the importance of prognosticators in determining the risk scores for patients. ERVs have the potential to supplement and improve existing prognostic models for determining risk of relapse in prostate cancer patients.

## 5. Conclusions

We identified the ERV expression differences in prostate cancer from patients of different ancestries to better understand the biological basis for PCa disparities. We identified ERVs and their nearby genes that had significant concordant expression differences between the two ancestries and determined the metabolic and immune pathways where these genes have important functions.

We built a BCR risk-prediction model using clinical data and ERV transcripts. We found that the combination of ERVs and clinical information outperforms prediction models based on clinical prognosticators alone. Measuring ERV expression may have the potential to help physicians to predict which patients would most benefit from active surveillance or radical therapy. Potentially, ERVs could also be of utility in clinically relevant prognostic models for other cancers. Ultimately, combining ERVs with other prognosticators, such as the ones identified by Long et al. [1], and with clinical parameters, may constitute an even more accurate method of identifying risk. In the future, experiments to knock out or overexpress ERVs in cell and tissue culture may further advance our understanding of the consequences of differential regulation of ERVs among people of different geographical ancestry.

## Figures and Tables

**Table 1:** Summary statistics for all samples filtered from GSE54460. Gleason value was assigned as shown below and ancestries were identified using LASER.

Characteristic	Training Set, *n* = 57 ^1^	Test Set, *n* = 35 ^1^
Gleason Value		
1 (<3)	3 (5.3%)	7 (20%)
2 (3 + 4)	36 (63%)	15 (43%)
3 (4 + 3)	10 (18%)	7 (20%)
4 (>7)	8 (14%)	6 (17%)
Age	62 (56, 65)	61 (57, 66)
Pre-PSA	7 (5, 13)	8 (6, 13)
Ancestry		
BA	23 (40%)	4 (11%)
WA	34 (60%)	31 (89%)
Tumor Stage		
1	0 (0%)	12 (34%)
2	45 (79%)	19 (54%)
3	11 (19%)	4 (11%)
4	1 (1.8%)	0 (0%)

^1^ n (%); Median (IQR).

## Data Availability

Data used for RNA-seq analysis can be found at GEO Accession GSE54460. The other datasets used/generated in this study are available from the corresponding author on reasonable request.

## Supplementary Materials

The following are available online at https://www.mdpi.com/article/10.3390/cancers13246347/s1, Table S1: Total sequencing summary, Table S2: Sample summary, Table S3: Differentially expressed ERVs and nearby genes, Table S4: Tissue microarray data for White Americans, Table S5: Tissue microarray data for Black Americans, Table S6: Cox proportional hazards model coefficients.
